# A mutational signature for colorectal cancer prognosis prediction: Associated with immune cell infiltration

**DOI:** 10.1002/ctm2.414

**Published:** 2021-05-06

**Authors:** Liang Xu, Yanyun Lin, Xijie Chen, Lisheng Zheng, Yufeng Cheng, Jiancong Hu, Bin Zheng, Bin Zhang, Guanman Li, Zengjie Chi, Shuang Guo, Danling Liu, Xiaosheng He, Ping Lan

**Affiliations:** ^1^ Guangdong Provincial Key Laboratory of Colorectal and Pelvic Floor Diseases Department of Colorectal Surgery Guangdong Institute of Gastroenterology The Sixth Affiliated Hospital Sun Yat‐sen University Guangzhou China; ^2^ State Key Laboratory of Oncology in South China Collaborative Innovation Center for Cancer Medicine Department of Clinical Laboratory Sun Yat‐sen University Cancer Center Guangzhou China

Dear Editor,

Colorectal cancer (CRC) is considered as a genetic disease, which arises from the stepwise accumulation of genetic and epigenetic alterations.^1^, ^2^ We found a novel mutational signature (MS) that could assist clinicians to select patients who are more suitable for immunotherapy; the risk score (RS) combined with pathological TNM stage could provide comprehensive and precise prognostic information for CRC patients. To explore the genomic basis of tumor variability in the tumor microenvironment of CRC, we integrated single nucleotide variation (SNV) and transcriptome data and collected information from 1133 and 588 CRC patients of the Memorial Sloan Kettering Cancer Center (MSKCC) and The Cancer Genome Atlas (TCGA) databases. In the training (MSKCC) cohort, we identified an MS consisting of 27 genomic variant genes and generated a prognostic model (Figures 1A and 1B). The date showed that the high‐risk group has poorer overall survival (OS), which was verified in both MSKCC and TCGA cohorts (Figures 1C and 1D). The Kaplan‐Meier survival curve and ROC curve were applied to evaluate the predicting power of the model by using R packages “survival” and “survival ROC.” The ROC curve indicated that the classifier had a good predictive ability (Figure 1E).The univariate and multivariate analyses revealed MS is an independent, unfavorable prognostic factor for CRCs (Figures 1F and 1G; Table S1).

**FIGURE 1:**
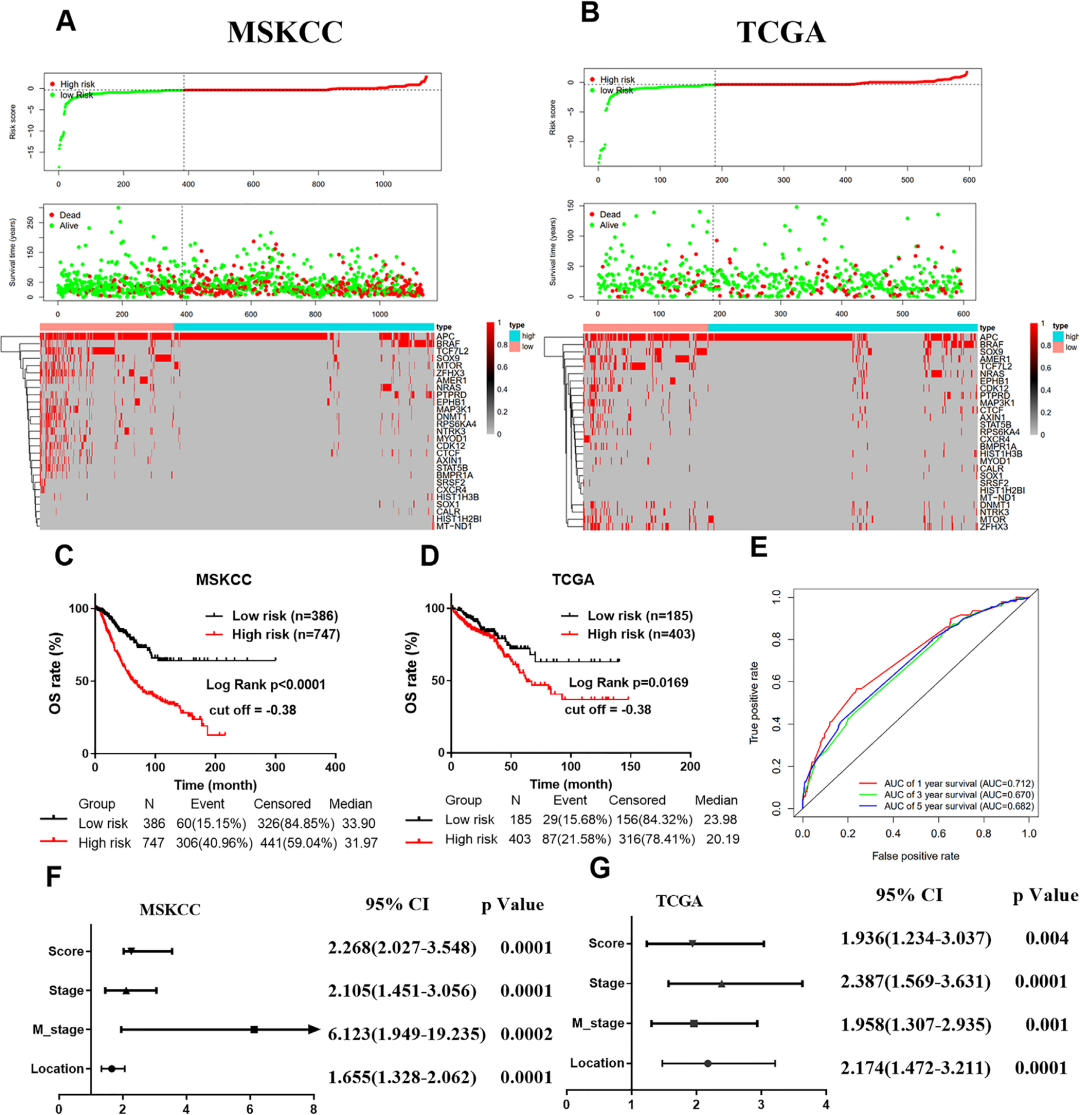
Identification of mutational signature and its prognostic value in CRC. (A and B) The distribution of risk score, survival duration, and status of patients, and a heatmap of mutated genes in the classifier. (C) Kaplan‐Meier curve for prognostic model showing the overall survival based on relative high‐ and low‐risk patients for OS in the training cohort. (D) Kaplan‐Meier curve for prognostic model showing the overall survival based on relative high‐ and low‐risk patients for OS in the validation cohort. (E) ROC curve analysis of the signature in 1‐year, 3‐year, and 5‐year in the MSKCC cohort. (F and G) Clinical pathologic features and mutational signature were selected for multivariate Cox regression analysis to build a predictive model for OS in MSKCC and TCGA. Abbreviation: AUC, area under the curve

A flowchart is shown in Figure S1. The coefficients of the 27 mutated genes are shown in Table S4. In the training cohort, a nomogram was generated to predict the OS of CRC patients (Figure S2A). The predictors included tumor location, M stage, TNM stage, and RS, among which the RS had the highest C‐index (Figures S2B and S2C). The clinical figures of CRC patients are listed in Table S2 and S3.

To explore the differences of genomic alterations in these two groups, we analyzed the data containing somatic mutations from the TCGA database. First, it revealed a significant enrichment of different mutations between low‐ and high‐risk groups (Figure 2A). The data showed that more than 90% of CRC in the low‐risk group had more mutations in the MSKCC cohort (Figure 2B), which means most of the genes with more mutations in the low‐risk group. Besides, the low‐ and high‐risk groups had different distribution of the top 10 mutated genes (Figure 2C). Significant enrichment of oncogenic alterations in such genes as BRAF, ZFHX3, and MTOR was found in right‐sided tumors and MSI (Microsatellite instability) patients, while oncogenic alteration of APC (Adenomatous Polyposis Coli) was primary found in the left‐sided tumors and MSS (Microsatellite stability) patients (Figure 2D). And all the results were consistent in the validation cohort (Figures 2E–2H; Figure S5).

**FIGURE 2:**
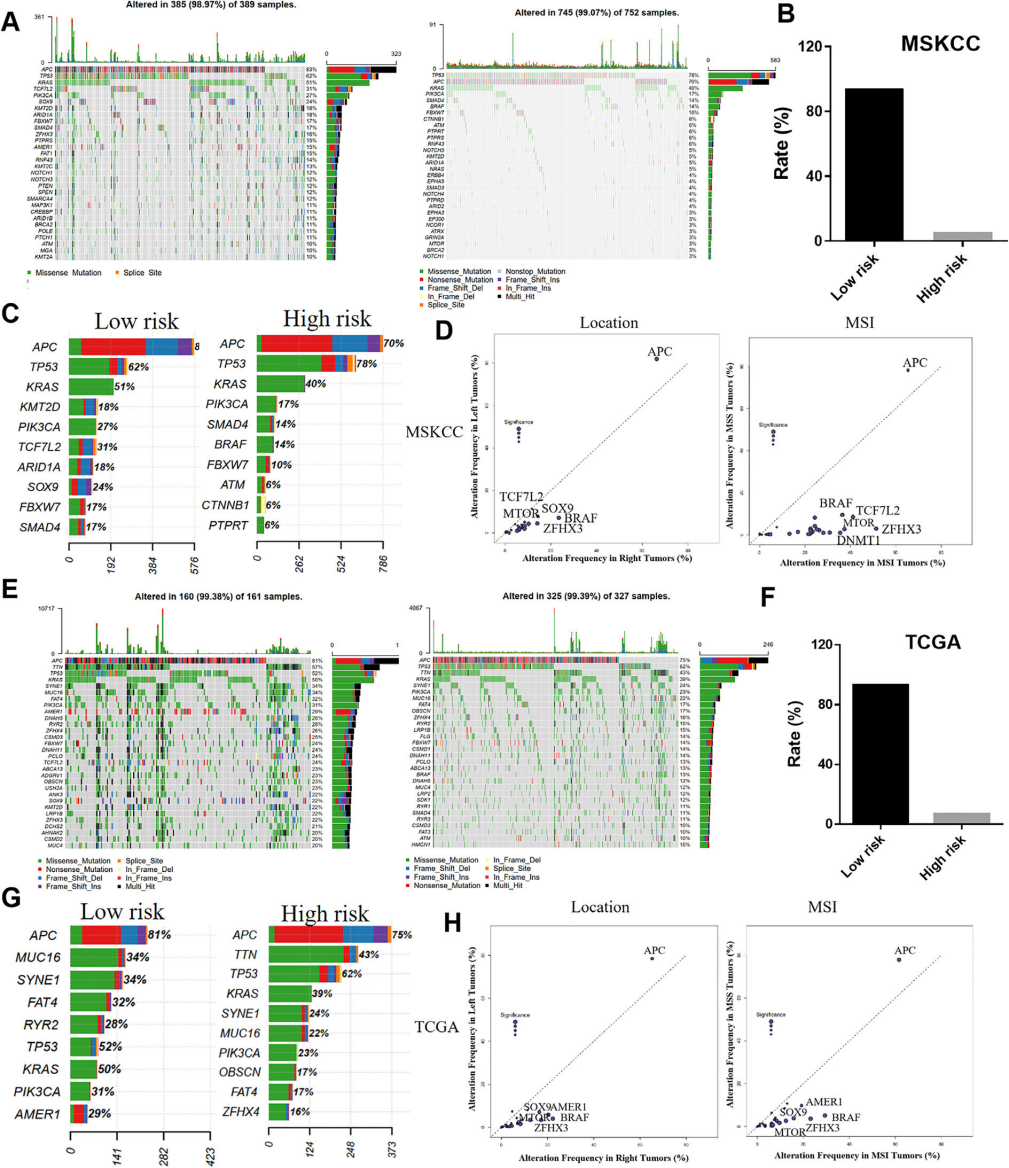
Mutational landscape of significantly mutated genes in the training cohort and verification cohort. (A) Top 30 genes with the most significant mutations in the MSKCC cohort. The bar chart above shows the total number of synonymous and non‐synonymous mutations in each patient's top 30 genes. The bar chart on the right shows the number of samples in which the 30 genes were mutated at low‐ and high‐risk groups. It is about 98.97% of patients have been detected to have genetic mutations. The different colors in the thermogram indicate the type of mutation; gray indicates no mutation. (B) The low‐risk group with more mutations, while the high‐risk group with fewer mutations in the MSKCC cohort. (C) The top 10 most high mutation genes in low‐ and high‐risk group in the MSKCC cohort. (D) Genomic alteration enrichment analysis by primary tumor site in location and molecular subtype in MSKCC cohort. (E) Top 30 genes with the most significant mutations in the TCGA cohort. The bar chart above shows the total number of synonymous and non‐synonymous mutations in each patient's top 30 genes. The bar chart on the right shows the number of samples in which the 30 genes were mutated at low‐ and high‐risk groups. It is about 99.38% of patients have been detected to have genetic mutations. The different colors in the thermogram indicate the type of mutation; gray indicates no mutation. (F) The low‐risk group with more mutations, while the high‐risk group with fewer mutations in the TCGA cohort. (G) The top 10 most high mutation genes in low‐ and high‐risk group in TCGA cohort. (H) Genomic alteration enrichment analysis by primary tumor site in location and molecular subtype in TCGA cohort

The MSI status is critical when considering immunotherapy and chemotherapeutic drugs as options for CRC patients.^3^, ^4^ The RS was observed to be significantly associated with the status of MSI/dMMR and other clinical features (Figures 3A and 3B; Figure S6). In line with previous observation, the status of MSI was more common in low‐risk group (Figure 3C). Furthermore, we observed that low‐risk group exhibited a higher mutations number (Figures 3D–3F). Due to the hypermutation or high mutational load, these patients might have increased neoantigens, leading to increased immune infiltration, and thus might be more sensitive to immunotherapy. We speculate that there is a potential connection between the MS model and the immune environment.

**FIGURE 3:**
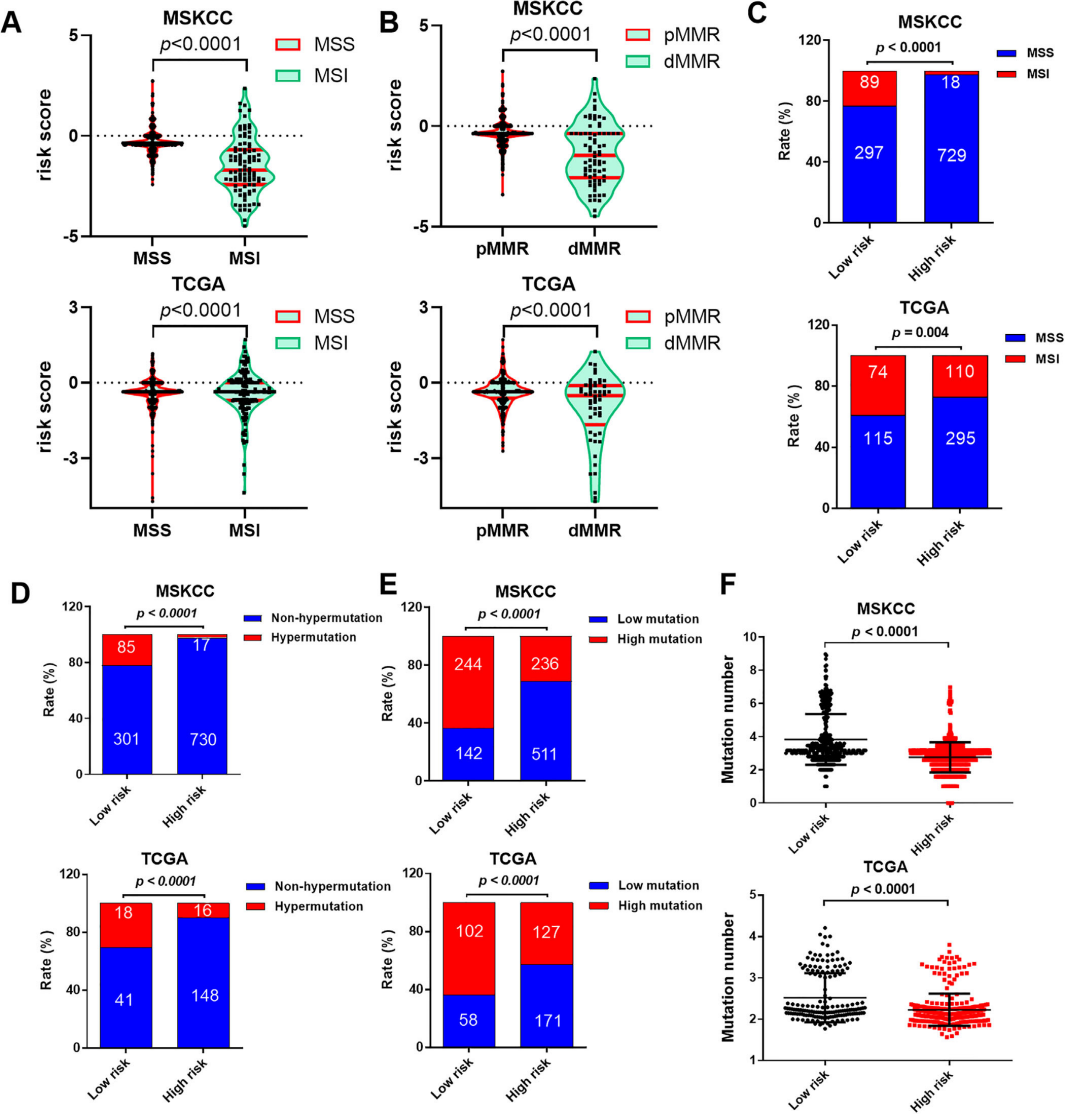
The mutational signature is associated with the genomic features of microsatellite instability (MSI) and mismatch repair (dMMR) in CRC. (A) MSI cancers have significantly lower risk score than the MSS cancers both in the training and validation cohorts. (B) The dMMR cancers have significantly lower risk score than the pMMR cancers both in the training and validation cohorts. (C) The proportion of MSI was significantly increased in the low‐risk group both in the training and validation cohorts. (D) The proportion of hypermutation was significantly increased in the low‐risk group both in the training and validation cohorts. (E) The proportion of high mutation was significantly increased in the low‐risk group both in the training and validation cohorts. (F) The low‐risk cancers have significantly higher mutation number than the high‐risk cancers both in the training and validation cohorts

To further clarify the relationship between MS and immune‐phenotyping, we analyzed SNV and transcriptome data in the TCGA database. The immune activity was determined by analyzing 29 immune‐related genesets. These genesets were analyzed using the ssGSEA.^5^ A heatmap of the infiltration levels and scores of each sample of immune cells in the three subtypes is shown in Figure 4A. A higher expression level of the PD‐L1 gene was found in the immunity‐H cluster, and the immunity‐H cluster was correlated with better survival outcome (Figures 4B and S5A‐S5D). The immunity‐H cluster was significantly enriched in the low‐risk group (Figure 4E). To reconfirm the findings above, we also performed consensus molecular subgroups (CMS) classification,^6^ which give a more profound biological insight into immunity typing, and has a strong prognostic effect. PD‐L1 was highly expressed in CMS1, which is defined by upregulation of immune genes and associated with MSI‐h. The data suggested that CMS1 cluster was significantly enriched in the low‐risk group (Figures 4D and 4F), and the CMS1 cluster is more likely benefit from PD‐L1 inhibitor treatment.

**FIGURE 4:**
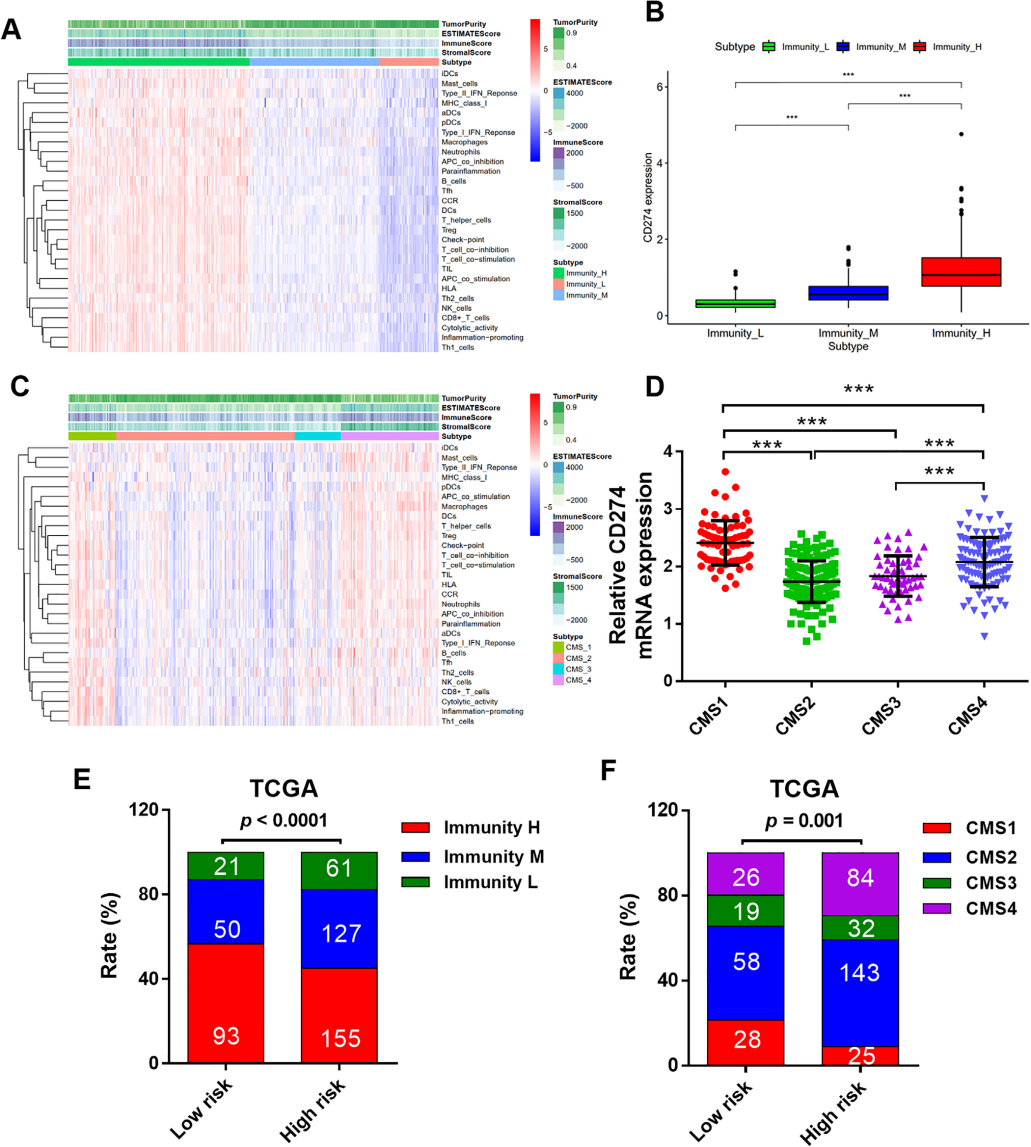
Mutational signature is associated with immune activity in CRC. (A) The immune cell infiltration level in each subtype, tumor purity, ESTIMATE score, stromal score, and immune score were evaluated by ESTIMATE. (B) Comparison of PD‐L1 (CD274) expression between CRC subtypes. (C) The immune cell infiltration level in each CMS subtype, tumor purity, ESTIMATE score, stromal score, and the immune score were evaluated by the ESTIMATE algorithm. (D) CD274 mRNA expression in CMS subtype. (E) The distribution of CRC subtypes in high‐ and low‐risk groups. (F) The distribution of CMS subtypes in high‐ and low‐risk groups

To further investigate the potential predictive value of the MS for the immune status, we examined the possible associations between the immune status and RS. TMB score was negatively correlated with the RS, but TMB was positively correlated with immune score (Figures S6A and S6B). Therefore, we postulated that the RS was negatively associated with immune score. We found a low immune score was related to a worse OS outcome (Figure S3C), and the low‐risk group may also be associated with a better survival. To figure out the infiltrated immune cell composition in the defined risk groups, we analyzed the expression signature matrix of 22 infiltrated immune cell types in tumor samples using the CIBERSORT test (Figures S3D‐S3G). Regarding tumor‐infiltrating immune cells in CRC microenvironment, the number of CD4 memory T cells decreased, and the macrophage M0 increased in the high‐risk group (Figures S6D‐S6G).

Immunotherapy has been raised as a novel effective treatment against CRC; however, the current guidelines only based on the TNM stage cannot reflect the information of host immune system response.^7^, ^8^ In clinic, MSI‐H is an especially good indicator for checkpoint blockade immunotherapy in CRC, but only about 45% of MSI‐H CRC patients could benefit from immunotherapy.^9^ In our prediction model, we have identified a novel MS, which can generate a prognostic tool to effectively classify CRC patients with different OS risks. Moreover, the MS classifier can be used to predict patients who are more suitable for immunotherapy, and a nomogram comprising the MS could help medical staff in directing personalized therapeutic treatment selection for CRC patients.

## CONFLICT OF INTEREST

The authors declare that there is no conflict of interest that could be perceived as prejudicing the impartiality of the research reported.

## AUTHOR CONTRIBUTIONS

Study design: Ping Lan, Xiaosheng He, and Liang Xu. Literature research: Yanyun Lin and Xijie Chen. Data acquisition: Liang Xu, Yanyun Lin, and Xijie Chen. Data analysis/interpretation: Yanyun Lin and Guanman Li. Statistical analysis: Xijie Chen and Zengjie Chi. Manuscript preparation: Liang Xu and Bin Zheng. Manuscript definition of intellectual content: Lisheng Zheng and Bin Zheng. Manuscript editing: Yufeng Cheng, Jiancong Hu, Shuang Guo, and Danling Liu.

## DATA ACCESS, RESPONSIBILITY, AND ANALYSIS

All data generated or analyzed during the present study are available via the corresponding author on reasonable request.

## Supporting information

FIGURE S1 Flowchart detailing the procedure of analyzing mutational signature and their correlation with clinical characteristics and immunity, as well as prognostic models of mutated genes.
FIGURE S2 Univariate and multivariate COX regression analyses of clinical factors and independence associated with prognosis/nomogram A to predict the risk of overall survival in CRC. (A) Nomogram to predict distant metastasis‐free survival. (B) Calibration curves of the nomogram to predict overall survival at 3 and 5 years in the MSKCC cohort. (C) Calibration curves of the nomogram to predict overall survival at 3 and 5 years in the TCGA cohort.
FIGURE S3 The mutation rate of low‐ and high‐risk group. (A) The proportion of right tumor was significantly increased in the low‐risk group both in the training and validation cohorts. (B) The proportion of stage III‐IV was significantly increased in the high‐risk group both in the training and validation cohorts. (C) The high‐risk group has a significantly higher risk score than the low‐risk group both in the training and validation cohorts.
FIGURE S4 The mutation rate of low‐ and high‐risk group. (A) The proportion of right tumor was significantly increased in the low‐risk group both in the training and validation cohorts. (B) The proportion of stage III‐IV was significantly increased in the high‐risk group both in the training and validation cohorts. (C) The high‐risk group has a significantly higher risk score than the low‐risk group both in the training and validation cohorts.
FIGURE S5 Composition of immune cell. (A) PCA analysis of the three clusters. (B) Comparison of the expression levels of HLA genes between CRC subtypes (ANOVA test). (C) Kaplan‐Meier analysis of three immunity clusters. (D) Comparison of the stromal score, immune score, ESTIMATE score, and tumor purity between CRC subtypes (Mann‐Whitney U test).
FIGURE S6 Composition of immune cells at low‐ and high‐risk tissues in the TCGA cohort. (A) Correlation analysis between risk score and TMB in the TCGA dataset. (B) Correlation analysis between immune score and TMB in the TCGA dataset. (C) Kaplan‐Meier analysis between low and high immune score. (D) Fractions of immune cells in 63 high‐risk and 63 low‐risk groups in the TCGA dataset. (E) Correlation of immune cells in the TCGA dataset. (F) Comparison of immune cells between high‐ and low‐risk groups in the TCGA dataset. (G) GSEA analysis of high‐ and low‐risk group.
FIGURE S7 DCA curve for MSKCC and TCGA cohorts. (A) DCA curve at 3 and 5 years in the MSKCC cohort. (B) Enriched GO terms in the “biological process” category. Different colors indicate a different function. (C) Enriched KEGG biological pathways.
Table S1 Univariate and multivariate COX regression analyses of clinical factors and independence associated with prognosis.
Table S2 Relationship between risk score and clinicopathological factors in the training, external validation cohorts.
Table S3 Clinical characteristics.
Table S4 Twenty‐seven mutated genes and its coefficient.
Table S5 Relationship between risk score and gene mutation in the training, external validation cohorts.
