# Supplementary figures and images for: A mutational signature for colorectal cancer prognosis prediction: Associated with immune cell infiltration
Source: Clin Transl Med. 2021 May 6;11(5):e414. doi: 10.1002/ctm2.414 (PMC8101534; doi:10.1002/ctm2.414)

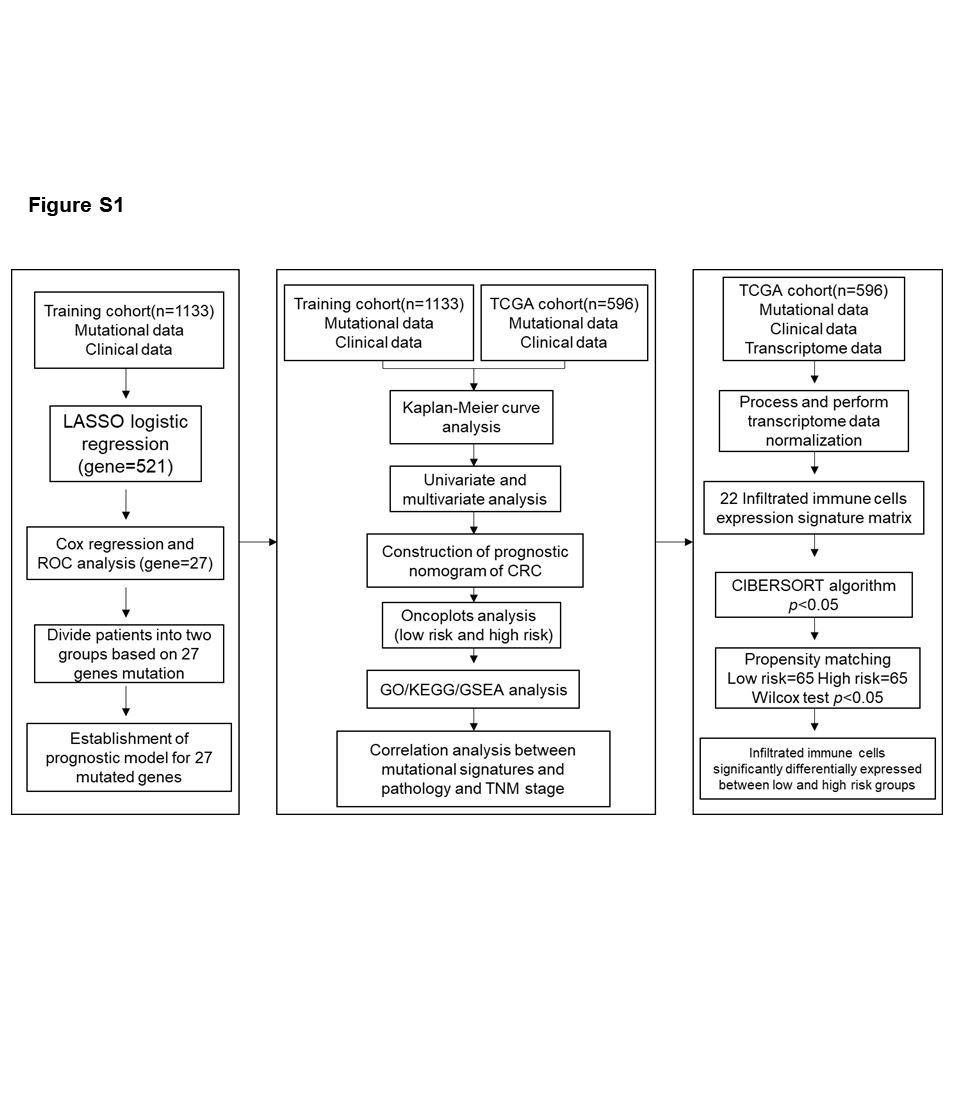

Supplement: Supplementary file 1 — FIGURE S1 Flowchart detailing the procedure of analyzing mutational signature and their correlation with clinical characteristics and immunity, as well as prognostic models of mutated genes. FIGURE S2 Univariate and multivariate COX regression analyses of clinical factors and independence associated with prognosis/nomogram A to predict the risk of overall survival in CRC. (A) Nomogram to predict distant metastasis‐free survival. (B) Calibration curves of the nomogram to predict overall survival at 3 and 5 years in the MSKCC cohort. (C) Calibration curves of the nomogram to predict overall survival at 3 and 5 years in the TCGA cohort. FIGURE S3 The mutation rate of low‐ and high‐risk group. (A) The proportion of right tumor was significantly increased in the low‐risk group both in the training and validation cohorts. (B) The proportion of stage III‐IV was significantly increased in the high‐risk group both in the training and validation cohorts. (C) The high‐risk group has a significantly higher risk score than the low‐risk group both in the training and validation cohorts. FIGURE S4 The mutation rate of low‐ and high‐risk group. (A) The proportion of right tumor was significantly increased in the low‐risk group both in the training and validation cohorts. (B) The proportion of stage III‐IV was significantly increased in the high‐risk group both in the training and validation cohorts. (C) The high‐risk group has a significantly higher risk score than the low‐risk group both in the training and validation cohorts. FIGURE S5 Composition of immune cell. (A) PCA analysis of the three clusters. (B) Comparison of the expression levels of HLA genes between CRC subtypes (ANOVA test). (C) Kaplan‐Meier analysis of three immunity clusters. (D) Comparison of the stromal score, immune score, ESTIMATE score, and tumor purity between CRC subtypes (Mann‐Whitney U test). FIGURE S6 Composition of immune cells at low‐ and high‐risk tissues in the TCGA cohort. (A) Correlation analysis b [file CTM2-11-e414-s001.zip › FigureS1.TIF]

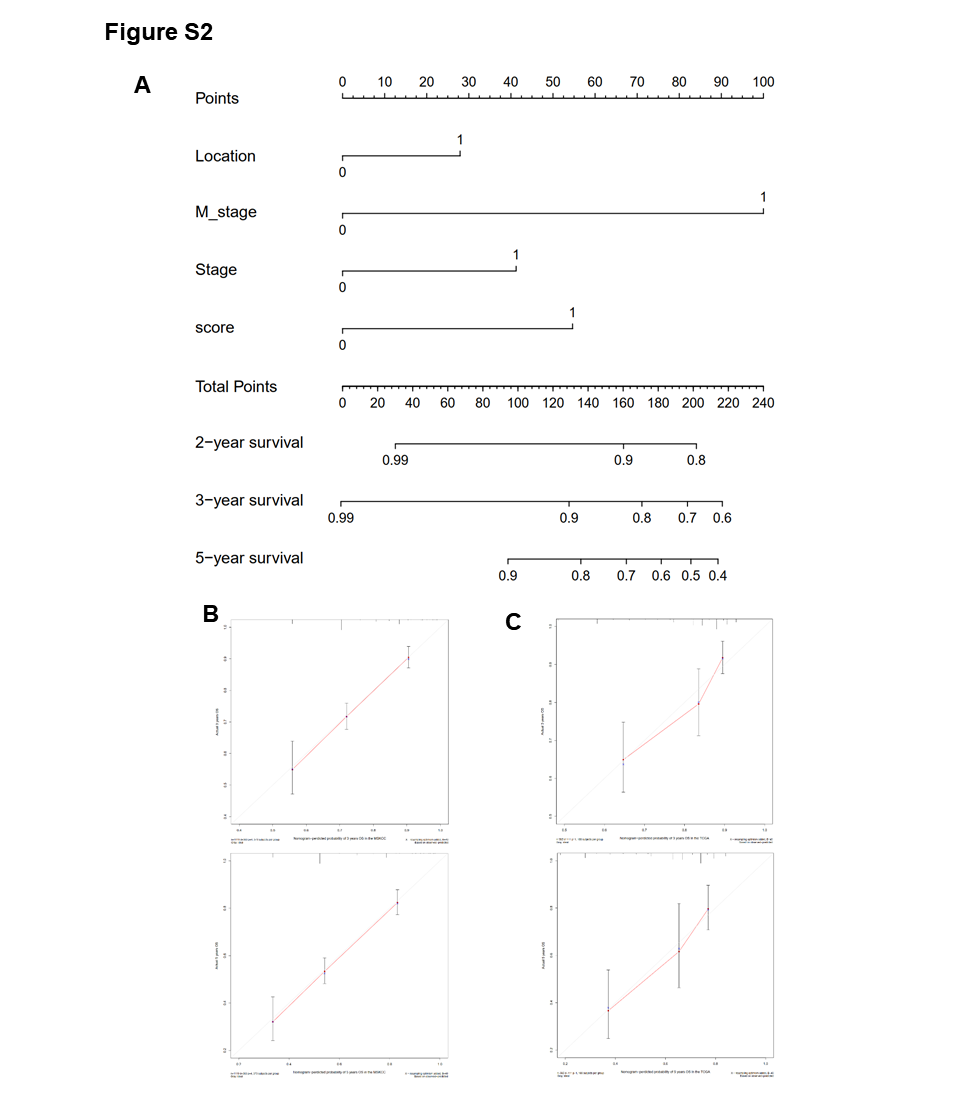

Supplement: Supplementary file 1 — FIGURE S1 Flowchart detailing the procedure of analyzing mutational signature and their correlation with clinical characteristics and immunity, as well as prognostic models of mutated genes. FIGURE S2 Univariate and multivariate COX regression analyses of clinical factors and independence associated with prognosis/nomogram A to predict the risk of overall survival in CRC. (A) Nomogram to predict distant metastasis‐free survival. (B) Calibration curves of the nomogram to predict overall survival at 3 and 5 years in the MSKCC cohort. (C) Calibration curves of the nomogram to predict overall survival at 3 and 5 years in the TCGA cohort. FIGURE S3 The mutation rate of low‐ and high‐risk group. (A) The proportion of right tumor was significantly increased in the low‐risk group both in the training and validation cohorts. (B) The proportion of stage III‐IV was significantly increased in the high‐risk group both in the training and validation cohorts. (C) The high‐risk group has a significantly higher risk score than the low‐risk group both in the training and validation cohorts. FIGURE S4 The mutation rate of low‐ and high‐risk group. (A) The proportion of right tumor was significantly increased in the low‐risk group both in the training and validation cohorts. (B) The proportion of stage III‐IV was significantly increased in the high‐risk group both in the training and validation cohorts. (C) The high‐risk group has a significantly higher risk score than the low‐risk group both in the training and validation cohorts. FIGURE S5 Composition of immune cell. (A) PCA analysis of the three clusters. (B) Comparison of the expression levels of HLA genes between CRC subtypes (ANOVA test). (C) Kaplan‐Meier analysis of three immunity clusters. (D) Comparison of the stromal score, immune score, ESTIMATE score, and tumor purity between CRC subtypes (Mann‐Whitney U test). FIGURE S6 Composition of immune cells at low‐ and high‐risk tissues in the TCGA cohort. (A) Correlation analysis b [file CTM2-11-e414-s001.zip › FigureS2.TIF]

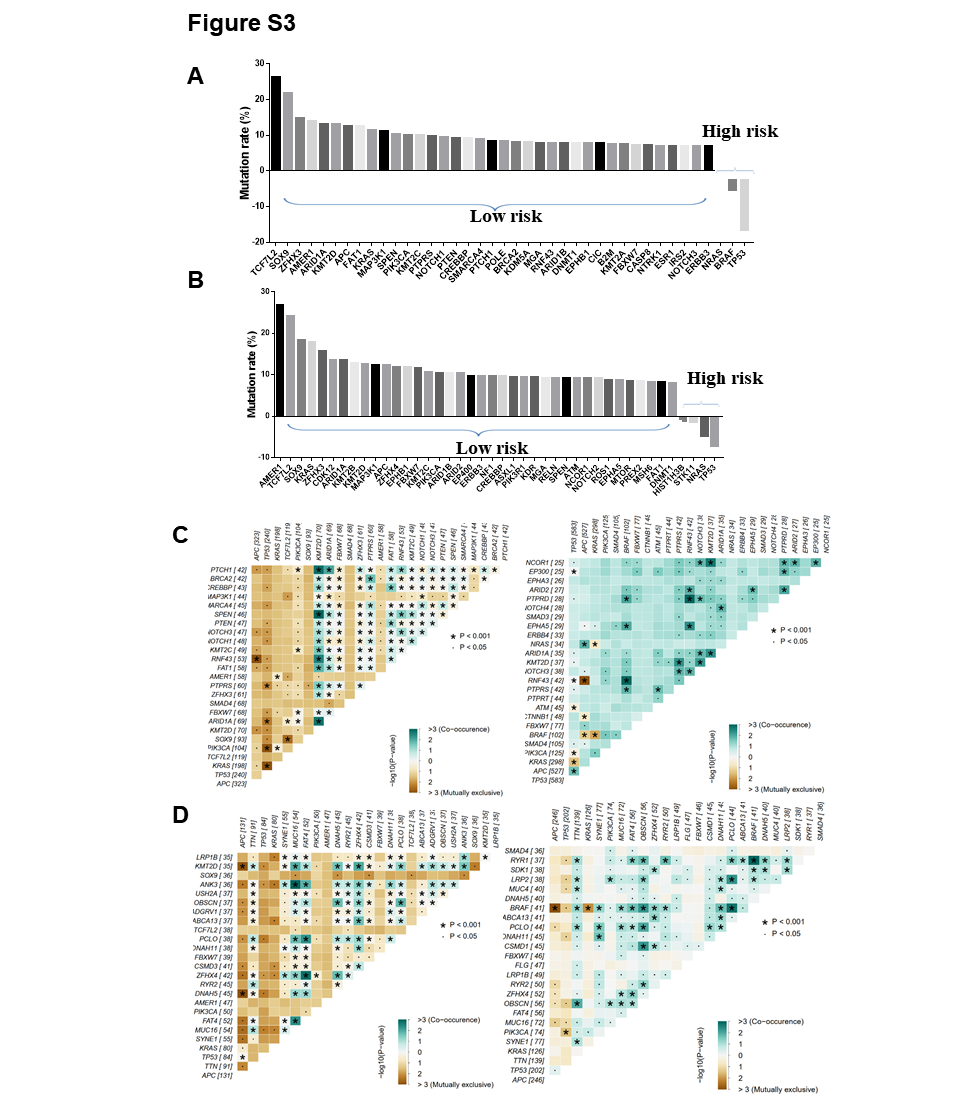

Supplement: Supplementary file 1 — FIGURE S1 Flowchart detailing the procedure of analyzing mutational signature and their correlation with clinical characteristics and immunity, as well as prognostic models of mutated genes. FIGURE S2 Univariate and multivariate COX regression analyses of clinical factors and independence associated with prognosis/nomogram A to predict the risk of overall survival in CRC. (A) Nomogram to predict distant metastasis‐free survival. (B) Calibration curves of the nomogram to predict overall survival at 3 and 5 years in the MSKCC cohort. (C) Calibration curves of the nomogram to predict overall survival at 3 and 5 years in the TCGA cohort. FIGURE S3 The mutation rate of low‐ and high‐risk group. (A) The proportion of right tumor was significantly increased in the low‐risk group both in the training and validation cohorts. (B) The proportion of stage III‐IV was significantly increased in the high‐risk group both in the training and validation cohorts. (C) The high‐risk group has a significantly higher risk score than the low‐risk group both in the training and validation cohorts. FIGURE S4 The mutation rate of low‐ and high‐risk group. (A) The proportion of right tumor was significantly increased in the low‐risk group both in the training and validation cohorts. (B) The proportion of stage III‐IV was significantly increased in the high‐risk group both in the training and validation cohorts. (C) The high‐risk group has a significantly higher risk score than the low‐risk group both in the training and validation cohorts. FIGURE S5 Composition of immune cell. (A) PCA analysis of the three clusters. (B) Comparison of the expression levels of HLA genes between CRC subtypes (ANOVA test). (C) Kaplan‐Meier analysis of three immunity clusters. (D) Comparison of the stromal score, immune score, ESTIMATE score, and tumor purity between CRC subtypes (Mann‐Whitney U test). FIGURE S6 Composition of immune cells at low‐ and high‐risk tissues in the TCGA cohort. (A) Correlation analysis b [file CTM2-11-e414-s001.zip › FigureS3.TIF]

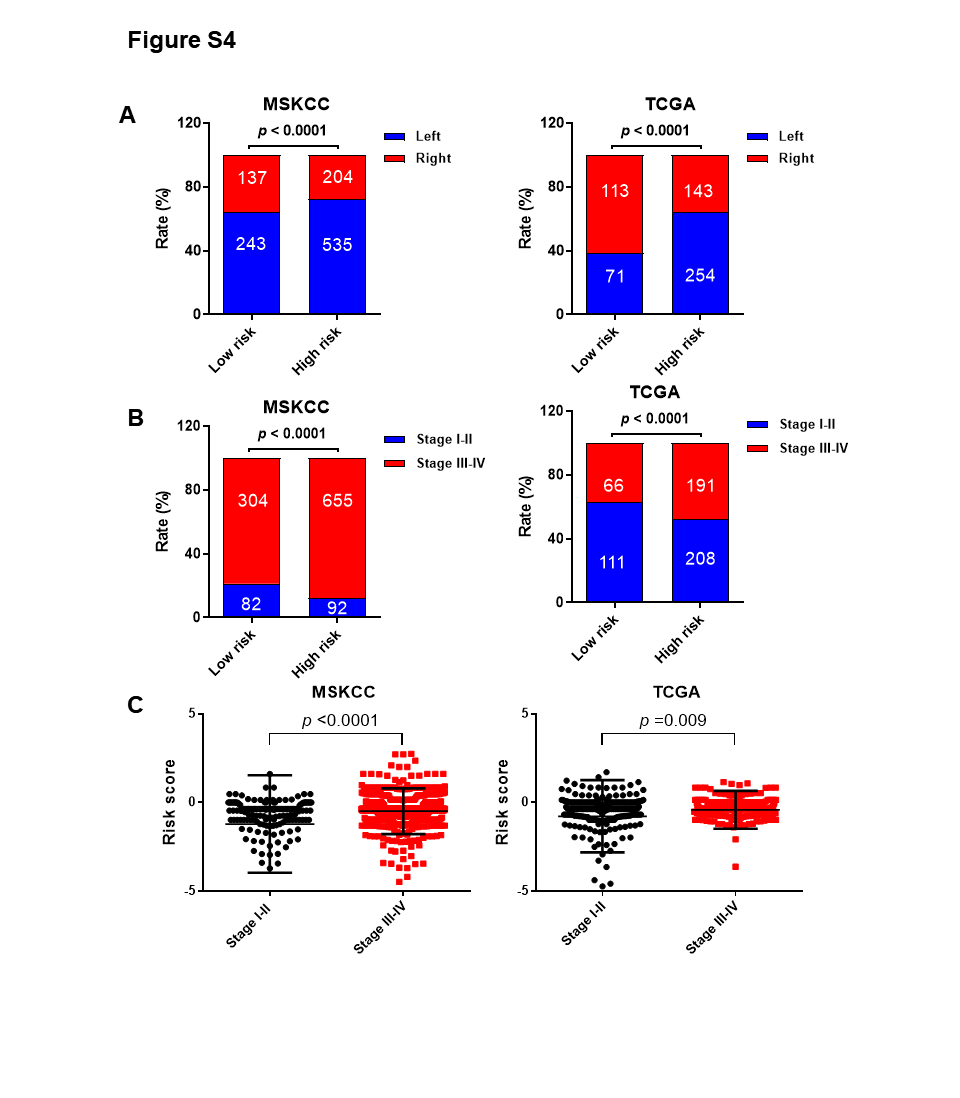

Supplement: Supplementary file 1 — FIGURE S1 Flowchart detailing the procedure of analyzing mutational signature and their correlation with clinical characteristics and immunity, as well as prognostic models of mutated genes. FIGURE S2 Univariate and multivariate COX regression analyses of clinical factors and independence associated with prognosis/nomogram A to predict the risk of overall survival in CRC. (A) Nomogram to predict distant metastasis‐free survival. (B) Calibration curves of the nomogram to predict overall survival at 3 and 5 years in the MSKCC cohort. (C) Calibration curves of the nomogram to predict overall survival at 3 and 5 years in the TCGA cohort. FIGURE S3 The mutation rate of low‐ and high‐risk group. (A) The proportion of right tumor was significantly increased in the low‐risk group both in the training and validation cohorts. (B) The proportion of stage III‐IV was significantly increased in the high‐risk group both in the training and validation cohorts. (C) The high‐risk group has a significantly higher risk score than the low‐risk group both in the training and validation cohorts. FIGURE S4 The mutation rate of low‐ and high‐risk group. (A) The proportion of right tumor was significantly increased in the low‐risk group both in the training and validation cohorts. (B) The proportion of stage III‐IV was significantly increased in the high‐risk group both in the training and validation cohorts. (C) The high‐risk group has a significantly higher risk score than the low‐risk group both in the training and validation cohorts. FIGURE S5 Composition of immune cell. (A) PCA analysis of the three clusters. (B) Comparison of the expression levels of HLA genes between CRC subtypes (ANOVA test). (C) Kaplan‐Meier analysis of three immunity clusters. (D) Comparison of the stromal score, immune score, ESTIMATE score, and tumor purity between CRC subtypes (Mann‐Whitney U test). FIGURE S6 Composition of immune cells at low‐ and high‐risk tissues in the TCGA cohort. (A) Correlation analysis b [file CTM2-11-e414-s001.zip › FigureS4.TIF]

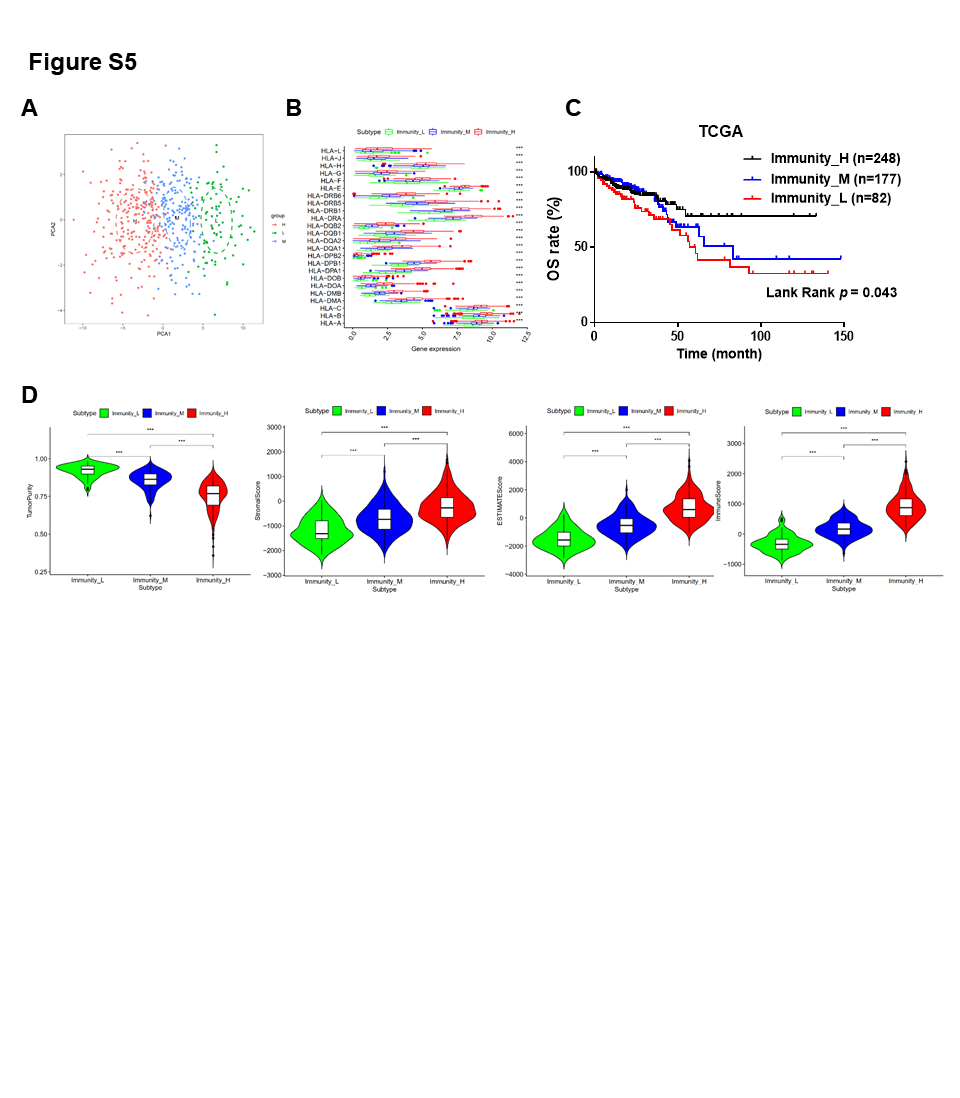

Supplement: Supplementary file 1 — FIGURE S1 Flowchart detailing the procedure of analyzing mutational signature and their correlation with clinical characteristics and immunity, as well as prognostic models of mutated genes. FIGURE S2 Univariate and multivariate COX regression analyses of clinical factors and independence associated with prognosis/nomogram A to predict the risk of overall survival in CRC. (A) Nomogram to predict distant metastasis‐free survival. (B) Calibration curves of the nomogram to predict overall survival at 3 and 5 years in the MSKCC cohort. (C) Calibration curves of the nomogram to predict overall survival at 3 and 5 years in the TCGA cohort. FIGURE S3 The mutation rate of low‐ and high‐risk group. (A) The proportion of right tumor was significantly increased in the low‐risk group both in the training and validation cohorts. (B) The proportion of stage III‐IV was significantly increased in the high‐risk group both in the training and validation cohorts. (C) The high‐risk group has a significantly higher risk score than the low‐risk group both in the training and validation cohorts. FIGURE S4 The mutation rate of low‐ and high‐risk group. (A) The proportion of right tumor was significantly increased in the low‐risk group both in the training and validation cohorts. (B) The proportion of stage III‐IV was significantly increased in the high‐risk group both in the training and validation cohorts. (C) The high‐risk group has a significantly higher risk score than the low‐risk group both in the training and validation cohorts. FIGURE S5 Composition of immune cell. (A) PCA analysis of the three clusters. (B) Comparison of the expression levels of HLA genes between CRC subtypes (ANOVA test). (C) Kaplan‐Meier analysis of three immunity clusters. (D) Comparison of the stromal score, immune score, ESTIMATE score, and tumor purity between CRC subtypes (Mann‐Whitney U test). FIGURE S6 Composition of immune cells at low‐ and high‐risk tissues in the TCGA cohort. (A) Correlation analysis b [file CTM2-11-e414-s001.zip › FigureS5.TIF]

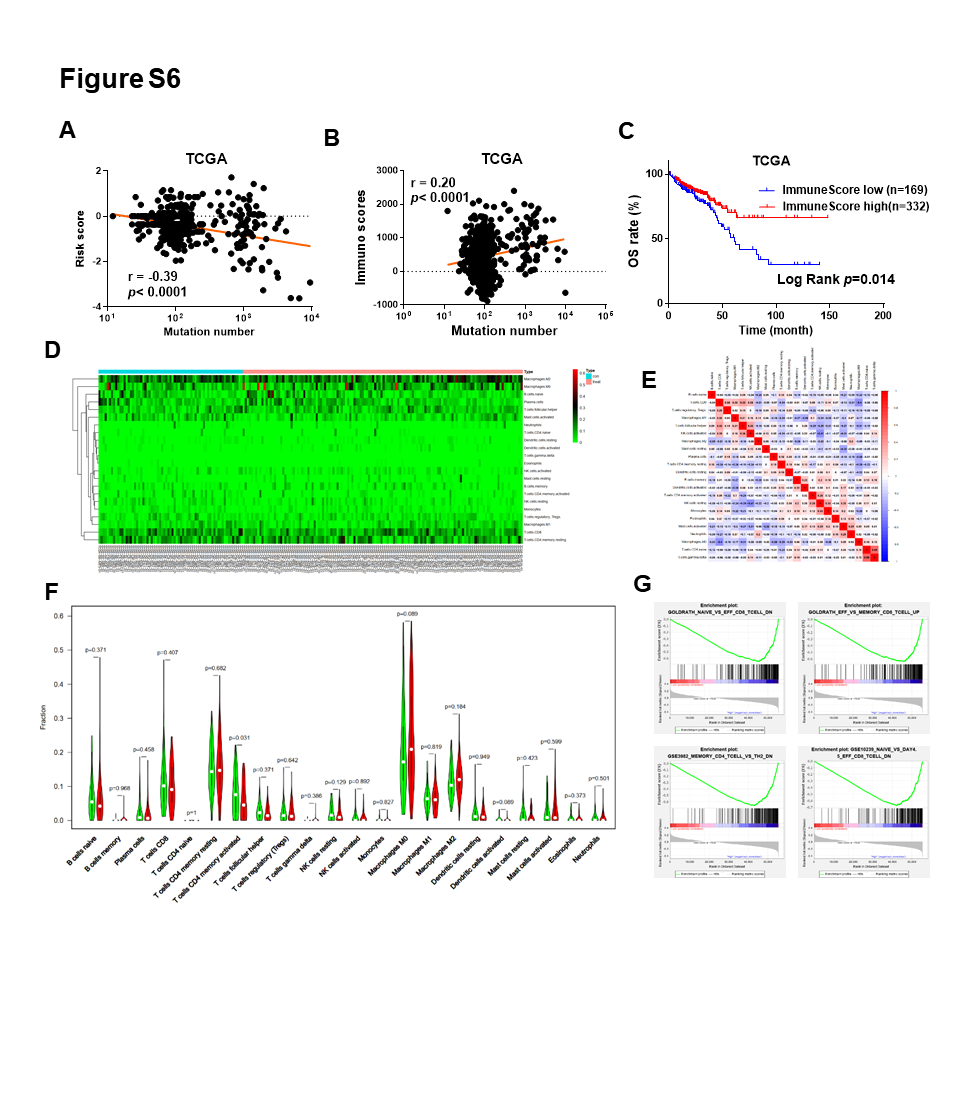

Supplement: Supplementary file 1 — FIGURE S1 Flowchart detailing the procedure of analyzing mutational signature and their correlation with clinical characteristics and immunity, as well as prognostic models of mutated genes. FIGURE S2 Univariate and multivariate COX regression analyses of clinical factors and independence associated with prognosis/nomogram A to predict the risk of overall survival in CRC. (A) Nomogram to predict distant metastasis‐free survival. (B) Calibration curves of the nomogram to predict overall survival at 3 and 5 years in the MSKCC cohort. (C) Calibration curves of the nomogram to predict overall survival at 3 and 5 years in the TCGA cohort. FIGURE S3 The mutation rate of low‐ and high‐risk group. (A) The proportion of right tumor was significantly increased in the low‐risk group both in the training and validation cohorts. (B) The proportion of stage III‐IV was significantly increased in the high‐risk group both in the training and validation cohorts. (C) The high‐risk group has a significantly higher risk score than the low‐risk group both in the training and validation cohorts. FIGURE S4 The mutation rate of low‐ and high‐risk group. (A) The proportion of right tumor was significantly increased in the low‐risk group both in the training and validation cohorts. (B) The proportion of stage III‐IV was significantly increased in the high‐risk group both in the training and validation cohorts. (C) The high‐risk group has a significantly higher risk score than the low‐risk group both in the training and validation cohorts. FIGURE S5 Composition of immune cell. (A) PCA analysis of the three clusters. (B) Comparison of the expression levels of HLA genes between CRC subtypes (ANOVA test). (C) Kaplan‐Meier analysis of three immunity clusters. (D) Comparison of the stromal score, immune score, ESTIMATE score, and tumor purity between CRC subtypes (Mann‐Whitney U test). FIGURE S6 Composition of immune cells at low‐ and high‐risk tissues in the TCGA cohort. (A) Correlation analysis b [file CTM2-11-e414-s001.zip › FigureS6.TIF]

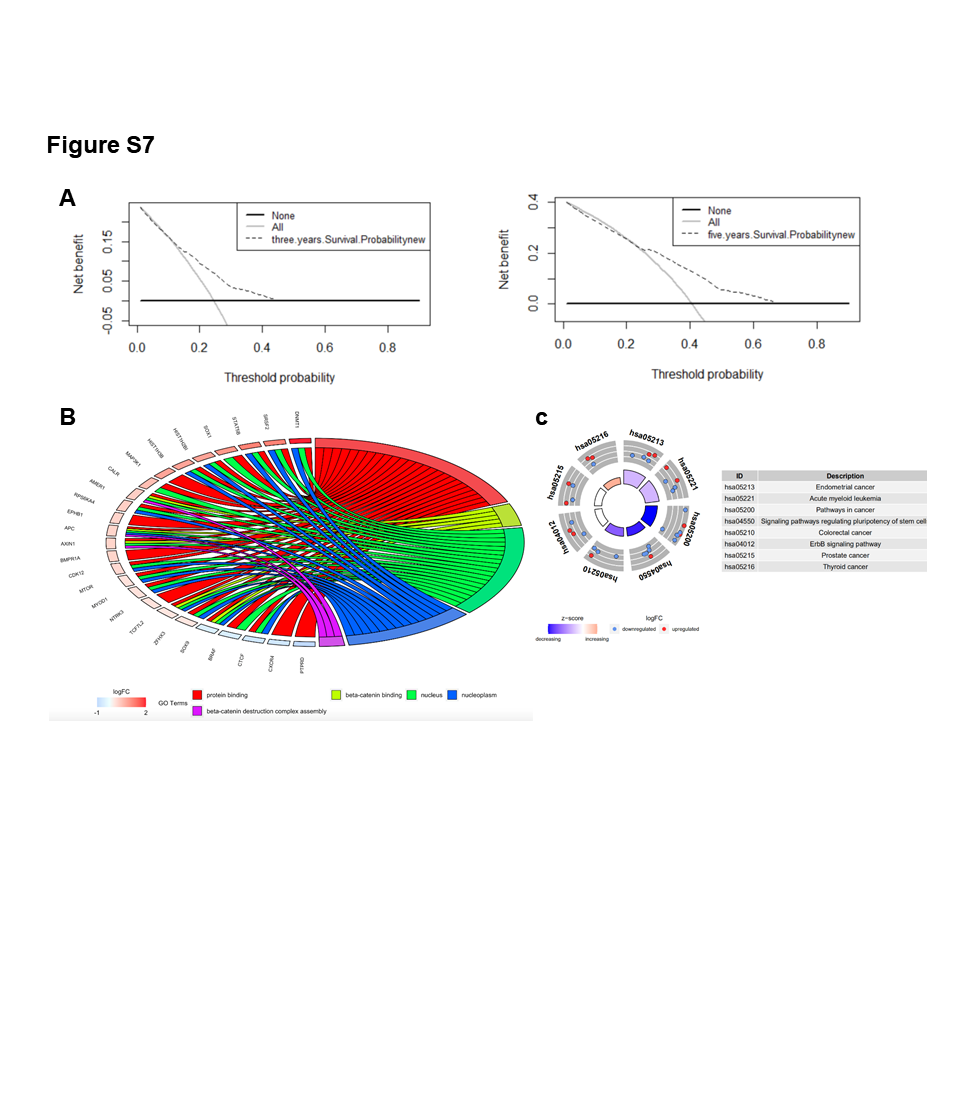

Supplement: Supplementary file 1 — FIGURE S1 Flowchart detailing the procedure of analyzing mutational signature and their correlation with clinical characteristics and immunity, as well as prognostic models of mutated genes. FIGURE S2 Univariate and multivariate COX regression analyses of clinical factors and independence associated with prognosis/nomogram A to predict the risk of overall survival in CRC. (A) Nomogram to predict distant metastasis‐free survival. (B) Calibration curves of the nomogram to predict overall survival at 3 and 5 years in the MSKCC cohort. (C) Calibration curves of the nomogram to predict overall survival at 3 and 5 years in the TCGA cohort. FIGURE S3 The mutation rate of low‐ and high‐risk group. (A) The proportion of right tumor was significantly increased in the low‐risk group both in the training and validation cohorts. (B) The proportion of stage III‐IV was significantly increased in the high‐risk group both in the training and validation cohorts. (C) The high‐risk group has a significantly higher risk score than the low‐risk group both in the training and validation cohorts. FIGURE S4 The mutation rate of low‐ and high‐risk group. (A) The proportion of right tumor was significantly increased in the low‐risk group both in the training and validation cohorts. (B) The proportion of stage III‐IV was significantly increased in the high‐risk group both in the training and validation cohorts. (C) The high‐risk group has a significantly higher risk score than the low‐risk group both in the training and validation cohorts. FIGURE S5 Composition of immune cell. (A) PCA analysis of the three clusters. (B) Comparison of the expression levels of HLA genes between CRC subtypes (ANOVA test). (C) Kaplan‐Meier analysis of three immunity clusters. (D) Comparison of the stromal score, immune score, ESTIMATE score, and tumor purity between CRC subtypes (Mann‐Whitney U test). FIGURE S6 Composition of immune cells at low‐ and high‐risk tissues in the TCGA cohort. (A) Correlation analysis b [file CTM2-11-e414-s001.zip › FigureS7.TIF]

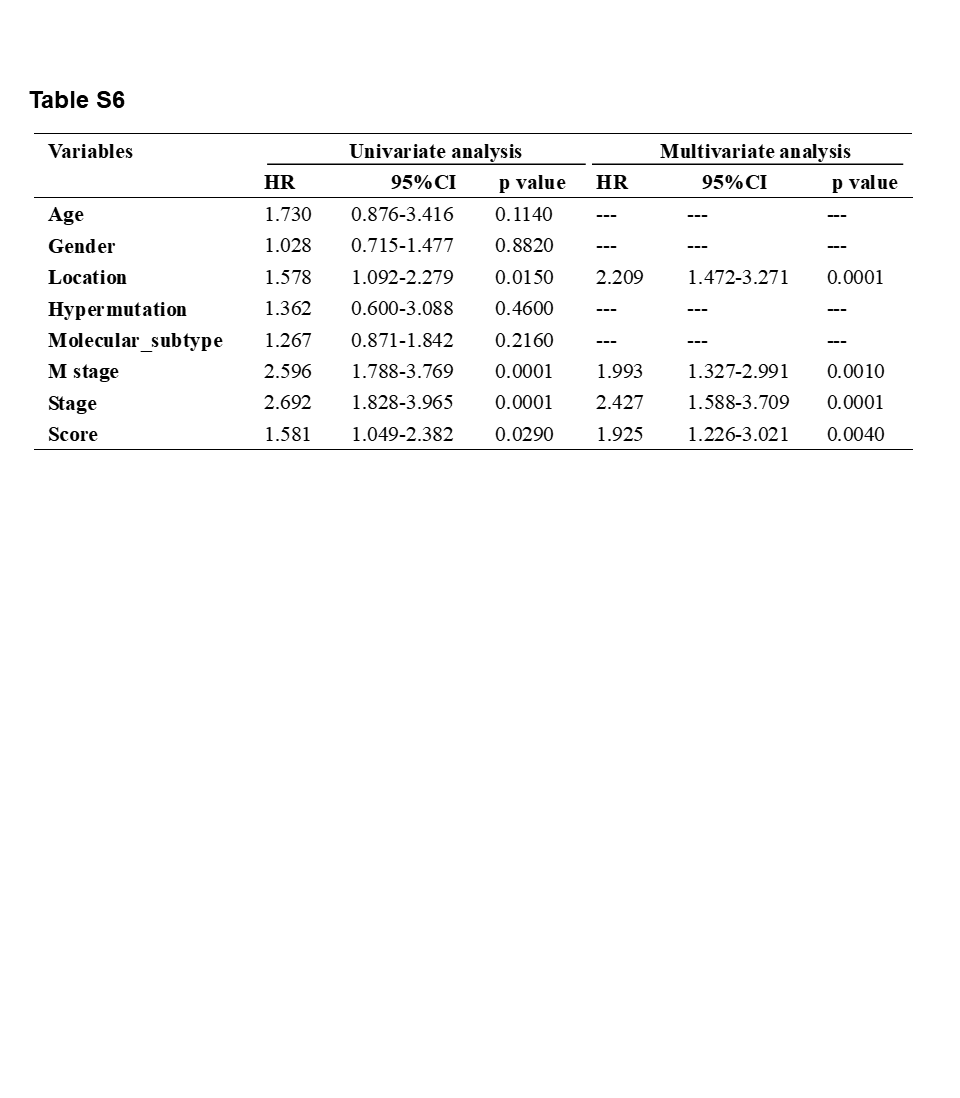

Supplement: Supplementary file 1 — FIGURE S1 Flowchart detailing the procedure of analyzing mutational signature and their correlation with clinical characteristics and immunity, as well as prognostic models of mutated genes. FIGURE S2 Univariate and multivariate COX regression analyses of clinical factors and independence associated with prognosis/nomogram A to predict the risk of overall survival in CRC. (A) Nomogram to predict distant metastasis‐free survival. (B) Calibration curves of the nomogram to predict overall survival at 3 and 5 years in the MSKCC cohort. (C) Calibration curves of the nomogram to predict overall survival at 3 and 5 years in the TCGA cohort. FIGURE S3 The mutation rate of low‐ and high‐risk group. (A) The proportion of right tumor was significantly increased in the low‐risk group both in the training and validation cohorts. (B) The proportion of stage III‐IV was significantly increased in the high‐risk group both in the training and validation cohorts. (C) The high‐risk group has a significantly higher risk score than the low‐risk group both in the training and validation cohorts. FIGURE S4 The mutation rate of low‐ and high‐risk group. (A) The proportion of right tumor was significantly increased in the low‐risk group both in the training and validation cohorts. (B) The proportion of stage III‐IV was significantly increased in the high‐risk group both in the training and validation cohorts. (C) The high‐risk group has a significantly higher risk score than the low‐risk group both in the training and validation cohorts. FIGURE S5 Composition of immune cell. (A) PCA analysis of the three clusters. (B) Comparison of the expression levels of HLA genes between CRC subtypes (ANOVA test). (C) Kaplan‐Meier analysis of three immunity clusters. (D) Comparison of the stromal score, immune score, ESTIMATE score, and tumor purity between CRC subtypes (Mann‐Whitney U test). FIGURE S6 Composition of immune cells at low‐ and high‐risk tissues in the TCGA cohort. (A) Correlation analysis b [file CTM2-11-e414-s001.zip › Table S6.TIF]

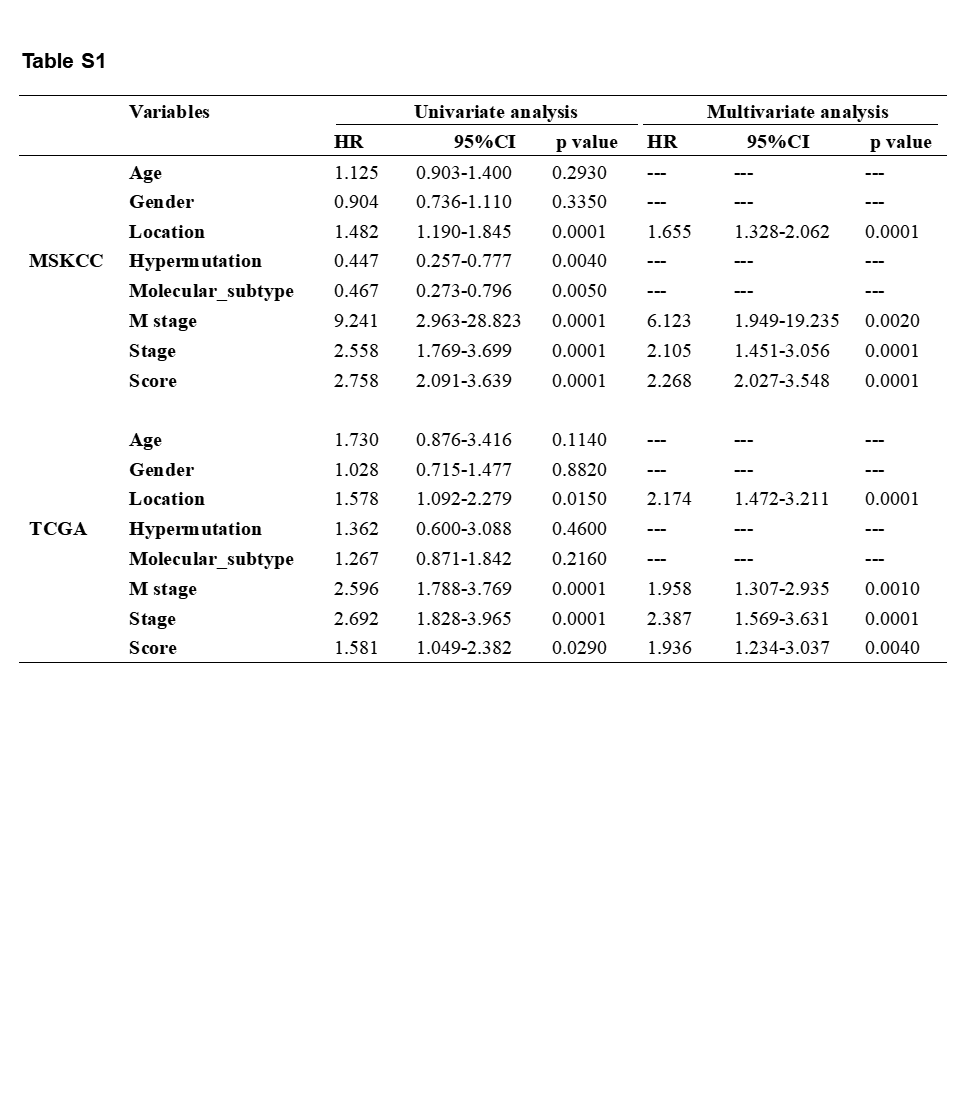

Supplement: Supplementary file 1 — FIGURE S1 Flowchart detailing the procedure of analyzing mutational signature and their correlation with clinical characteristics and immunity, as well as prognostic models of mutated genes. FIGURE S2 Univariate and multivariate COX regression analyses of clinical factors and independence associated with prognosis/nomogram A to predict the risk of overall survival in CRC. (A) Nomogram to predict distant metastasis‐free survival. (B) Calibration curves of the nomogram to predict overall survival at 3 and 5 years in the MSKCC cohort. (C) Calibration curves of the nomogram to predict overall survival at 3 and 5 years in the TCGA cohort. FIGURE S3 The mutation rate of low‐ and high‐risk group. (A) The proportion of right tumor was significantly increased in the low‐risk group both in the training and validation cohorts. (B) The proportion of stage III‐IV was significantly increased in the high‐risk group both in the training and validation cohorts. (C) The high‐risk group has a significantly higher risk score than the low‐risk group both in the training and validation cohorts. FIGURE S4 The mutation rate of low‐ and high‐risk group. (A) The proportion of right tumor was significantly increased in the low‐risk group both in the training and validation cohorts. (B) The proportion of stage III‐IV was significantly increased in the high‐risk group both in the training and validation cohorts. (C) The high‐risk group has a significantly higher risk score than the low‐risk group both in the training and validation cohorts. FIGURE S5 Composition of immune cell. (A) PCA analysis of the three clusters. (B) Comparison of the expression levels of HLA genes between CRC subtypes (ANOVA test). (C) Kaplan‐Meier analysis of three immunity clusters. (D) Comparison of the stromal score, immune score, ESTIMATE score, and tumor purity between CRC subtypes (Mann‐Whitney U test). FIGURE S6 Composition of immune cells at low‐ and high‐risk tissues in the TCGA cohort. (A) Correlation analysis b [file CTM2-11-e414-s001.zip › TableS1.TIF]

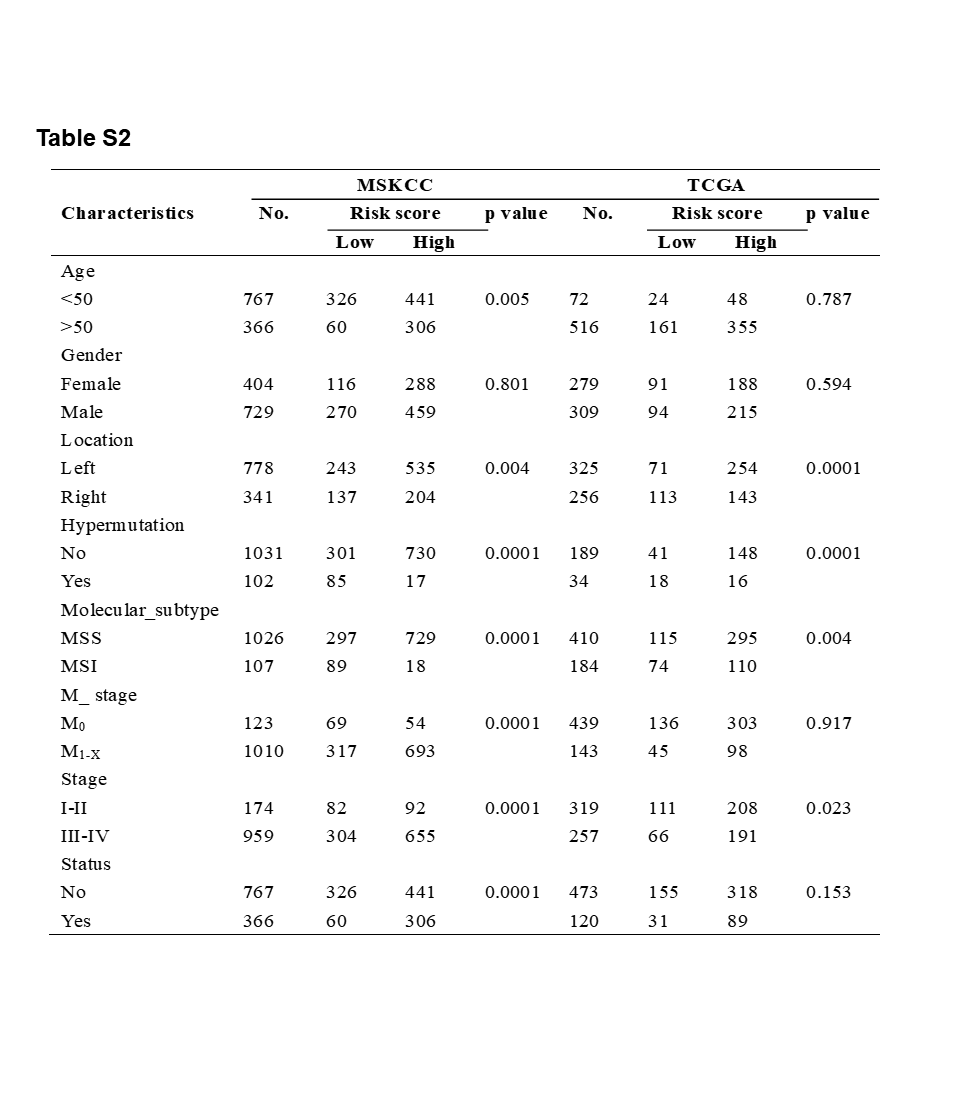

Supplement: Supplementary file 1 — FIGURE S1 Flowchart detailing the procedure of analyzing mutational signature and their correlation with clinical characteristics and immunity, as well as prognostic models of mutated genes. FIGURE S2 Univariate and multivariate COX regression analyses of clinical factors and independence associated with prognosis/nomogram A to predict the risk of overall survival in CRC. (A) Nomogram to predict distant metastasis‐free survival. (B) Calibration curves of the nomogram to predict overall survival at 3 and 5 years in the MSKCC cohort. (C) Calibration curves of the nomogram to predict overall survival at 3 and 5 years in the TCGA cohort. FIGURE S3 The mutation rate of low‐ and high‐risk group. (A) The proportion of right tumor was significantly increased in the low‐risk group both in the training and validation cohorts. (B) The proportion of stage III‐IV was significantly increased in the high‐risk group both in the training and validation cohorts. (C) The high‐risk group has a significantly higher risk score than the low‐risk group both in the training and validation cohorts. FIGURE S4 The mutation rate of low‐ and high‐risk group. (A) The proportion of right tumor was significantly increased in the low‐risk group both in the training and validation cohorts. (B) The proportion of stage III‐IV was significantly increased in the high‐risk group both in the training and validation cohorts. (C) The high‐risk group has a significantly higher risk score than the low‐risk group both in the training and validation cohorts. FIGURE S5 Composition of immune cell. (A) PCA analysis of the three clusters. (B) Comparison of the expression levels of HLA genes between CRC subtypes (ANOVA test). (C) Kaplan‐Meier analysis of three immunity clusters. (D) Comparison of the stromal score, immune score, ESTIMATE score, and tumor purity between CRC subtypes (Mann‐Whitney U test). FIGURE S6 Composition of immune cells at low‐ and high‐risk tissues in the TCGA cohort. (A) Correlation analysis b [file CTM2-11-e414-s001.zip › TableS2.TIF]

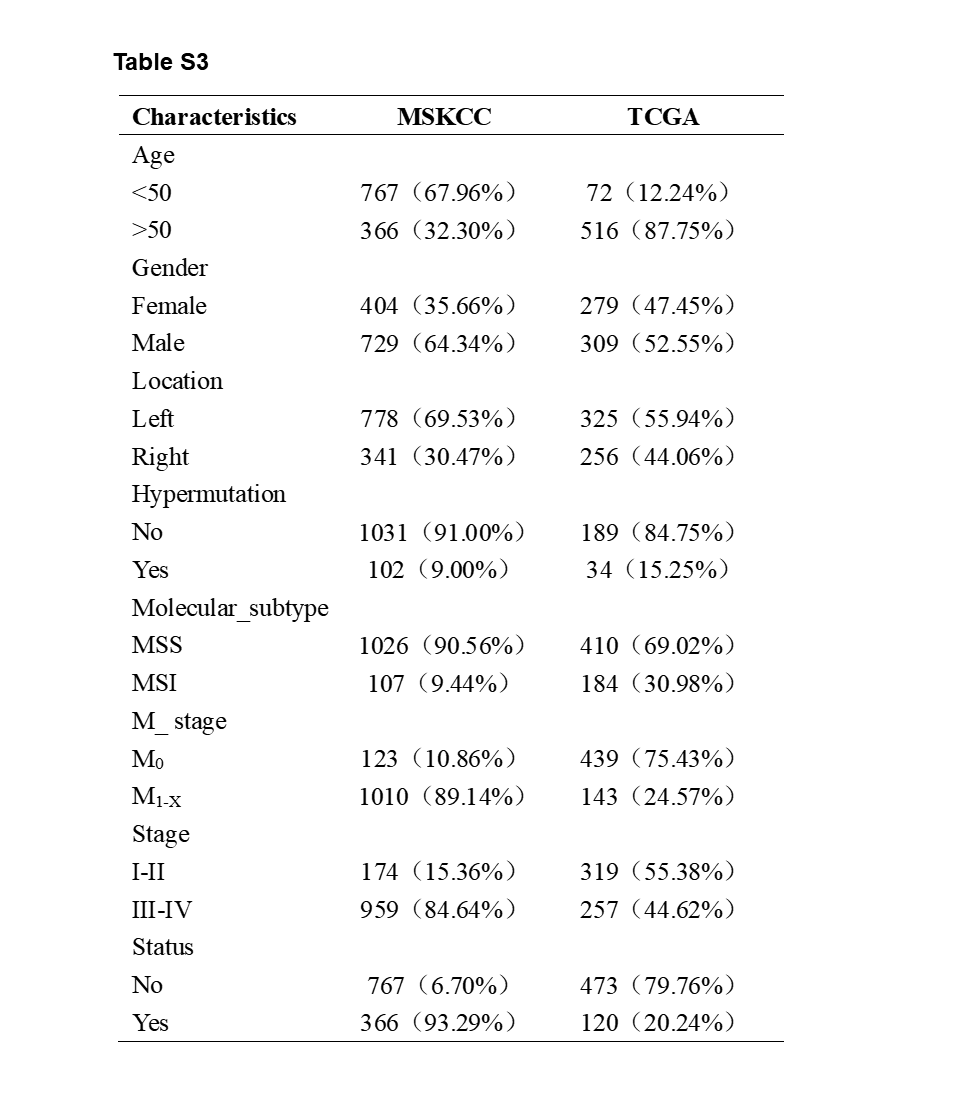

Supplement: Supplementary file 1 — FIGURE S1 Flowchart detailing the procedure of analyzing mutational signature and their correlation with clinical characteristics and immunity, as well as prognostic models of mutated genes. FIGURE S2 Univariate and multivariate COX regression analyses of clinical factors and independence associated with prognosis/nomogram A to predict the risk of overall survival in CRC. (A) Nomogram to predict distant metastasis‐free survival. (B) Calibration curves of the nomogram to predict overall survival at 3 and 5 years in the MSKCC cohort. (C) Calibration curves of the nomogram to predict overall survival at 3 and 5 years in the TCGA cohort. FIGURE S3 The mutation rate of low‐ and high‐risk group. (A) The proportion of right tumor was significantly increased in the low‐risk group both in the training and validation cohorts. (B) The proportion of stage III‐IV was significantly increased in the high‐risk group both in the training and validation cohorts. (C) The high‐risk group has a significantly higher risk score than the low‐risk group both in the training and validation cohorts. FIGURE S4 The mutation rate of low‐ and high‐risk group. (A) The proportion of right tumor was significantly increased in the low‐risk group both in the training and validation cohorts. (B) The proportion of stage III‐IV was significantly increased in the high‐risk group both in the training and validation cohorts. (C) The high‐risk group has a significantly higher risk score than the low‐risk group both in the training and validation cohorts. FIGURE S5 Composition of immune cell. (A) PCA analysis of the three clusters. (B) Comparison of the expression levels of HLA genes between CRC subtypes (ANOVA test). (C) Kaplan‐Meier analysis of three immunity clusters. (D) Comparison of the stromal score, immune score, ESTIMATE score, and tumor purity between CRC subtypes (Mann‐Whitney U test). FIGURE S6 Composition of immune cells at low‐ and high‐risk tissues in the TCGA cohort. (A) Correlation analysis b [file CTM2-11-e414-s001.zip › TableS3.TIF]

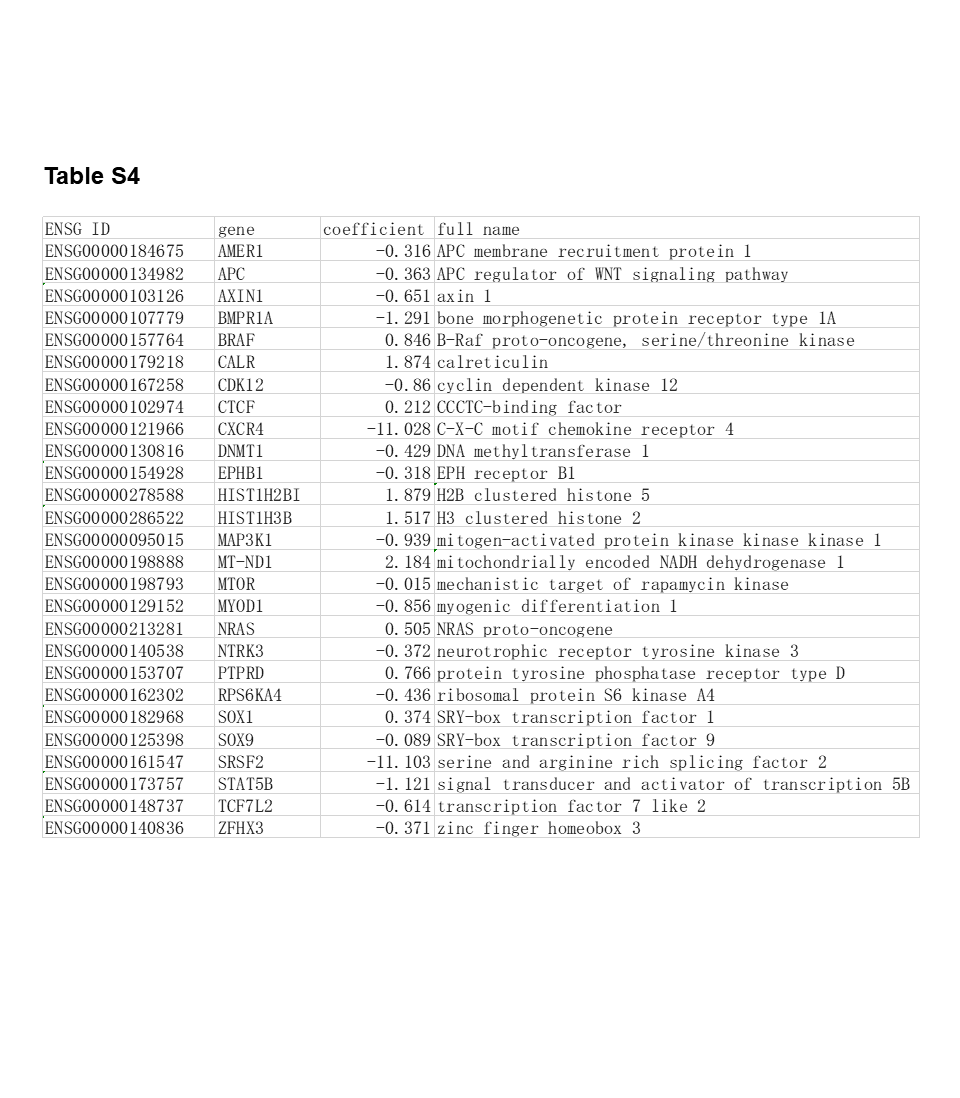

Supplement: Supplementary file 1 — FIGURE S1 Flowchart detailing the procedure of analyzing mutational signature and their correlation with clinical characteristics and immunity, as well as prognostic models of mutated genes. FIGURE S2 Univariate and multivariate COX regression analyses of clinical factors and independence associated with prognosis/nomogram A to predict the risk of overall survival in CRC. (A) Nomogram to predict distant metastasis‐free survival. (B) Calibration curves of the nomogram to predict overall survival at 3 and 5 years in the MSKCC cohort. (C) Calibration curves of the nomogram to predict overall survival at 3 and 5 years in the TCGA cohort. FIGURE S3 The mutation rate of low‐ and high‐risk group. (A) The proportion of right tumor was significantly increased in the low‐risk group both in the training and validation cohorts. (B) The proportion of stage III‐IV was significantly increased in the high‐risk group both in the training and validation cohorts. (C) The high‐risk group has a significantly higher risk score than the low‐risk group both in the training and validation cohorts. FIGURE S4 The mutation rate of low‐ and high‐risk group. (A) The proportion of right tumor was significantly increased in the low‐risk group both in the training and validation cohorts. (B) The proportion of stage III‐IV was significantly increased in the high‐risk group both in the training and validation cohorts. (C) The high‐risk group has a significantly higher risk score than the low‐risk group both in the training and validation cohorts. FIGURE S5 Composition of immune cell. (A) PCA analysis of the three clusters. (B) Comparison of the expression levels of HLA genes between CRC subtypes (ANOVA test). (C) Kaplan‐Meier analysis of three immunity clusters. (D) Comparison of the stromal score, immune score, ESTIMATE score, and tumor purity between CRC subtypes (Mann‐Whitney U test). FIGURE S6 Composition of immune cells at low‐ and high‐risk tissues in the TCGA cohort. (A) Correlation analysis b [file CTM2-11-e414-s001.zip › TableS4.TIF]

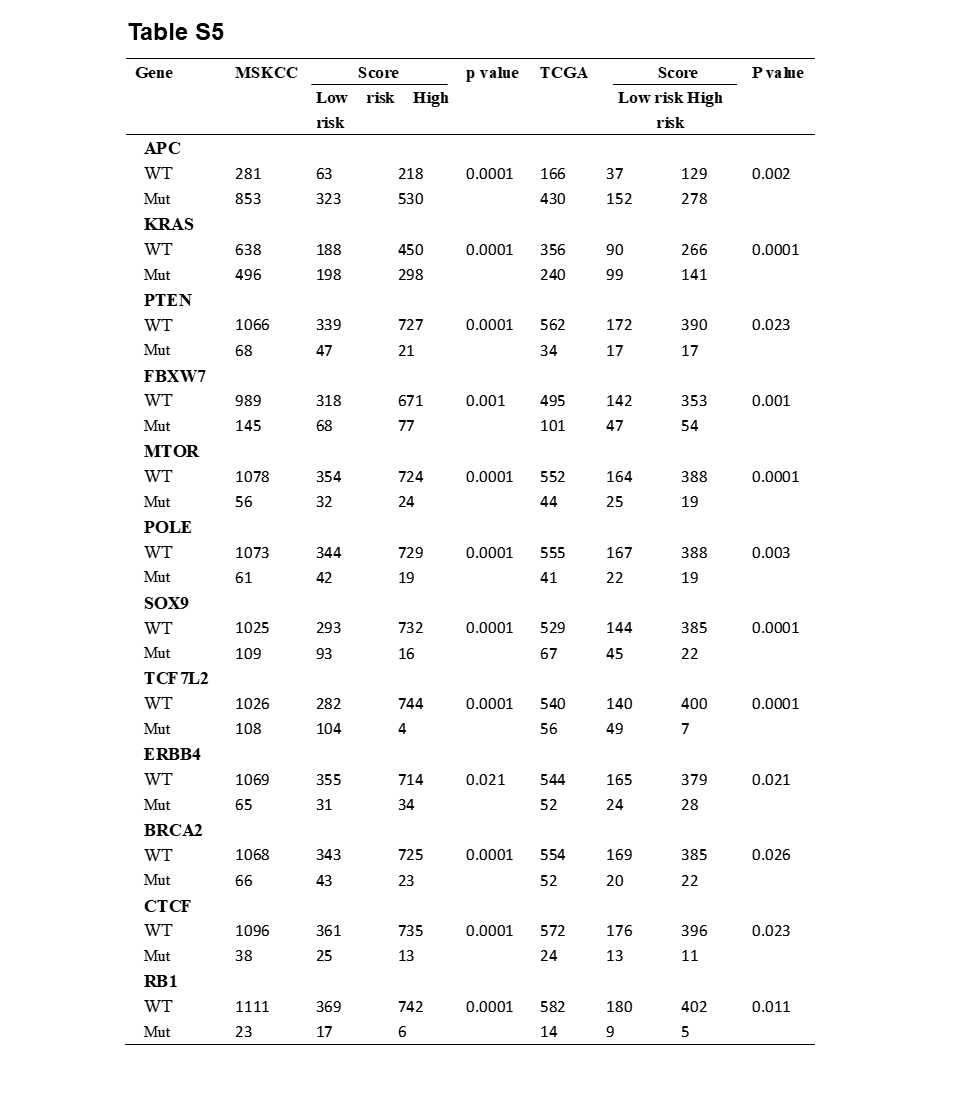

Supplement: Supplementary file 1 — FIGURE S1 Flowchart detailing the procedure of analyzing mutational signature and their correlation with clinical characteristics and immunity, as well as prognostic models of mutated genes. FIGURE S2 Univariate and multivariate COX regression analyses of clinical factors and independence associated with prognosis/nomogram A to predict the risk of overall survival in CRC. (A) Nomogram to predict distant metastasis‐free survival. (B) Calibration curves of the nomogram to predict overall survival at 3 and 5 years in the MSKCC cohort. (C) Calibration curves of the nomogram to predict overall survival at 3 and 5 years in the TCGA cohort. FIGURE S3 The mutation rate of low‐ and high‐risk group. (A) The proportion of right tumor was significantly increased in the low‐risk group both in the training and validation cohorts. (B) The proportion of stage III‐IV was significantly increased in the high‐risk group both in the training and validation cohorts. (C) The high‐risk group has a significantly higher risk score than the low‐risk group both in the training and validation cohorts. FIGURE S4 The mutation rate of low‐ and high‐risk group. (A) The proportion of right tumor was significantly increased in the low‐risk group both in the training and validation cohorts. (B) The proportion of stage III‐IV was significantly increased in the high‐risk group both in the training and validation cohorts. (C) The high‐risk group has a significantly higher risk score than the low‐risk group both in the training and validation cohorts. FIGURE S5 Composition of immune cell. (A) PCA analysis of the three clusters. (B) Comparison of the expression levels of HLA genes between CRC subtypes (ANOVA test). (C) Kaplan‐Meier analysis of three immunity clusters. (D) Comparison of the stromal score, immune score, ESTIMATE score, and tumor purity between CRC subtypes (Mann‐Whitney U test). FIGURE S6 Composition of immune cells at low‐ and high‐risk tissues in the TCGA cohort. (A) Correlation analysis b [file CTM2-11-e414-s001.zip › TableS5.TIF]
